# Alcohol and Illicit Drug Use Are Important Factors for School-Related Problems among Adolescents

**DOI:** 10.3389/fpsyg.2017.01023

**Published:** 2017-06-20

**Authors:** Ove Heradstveit, Jens C. Skogen, Jørn Hetland, Mari Hysing

**Affiliations:** ^1^Center for Alcohol and Drug Research, Stavanger University HospitalStavanger, Norway; ^2^Regional Centre for Child and Youth Mental Health and Child Welfare, Uni Research HealthBergen, Norway; ^3^Department of Health Promotion, Norwegian Institute of Public HealthBergen, Norway; ^4^Department of Psychosocial Science, University of BergenBergen, Norway

**Keywords:** alcohol use, illicit drug use, alcohol and drug-related problems, school-related problems, grade point average (GPA), school attendance

## Abstract

The aim of this study was to investigate the association between alcohol and drug use, and school-related problems measured by low grade point average (GPA) and high school attendance. We also examined potential confounding effects from mental health problems. Although the issue is not new within current literature, the present study has its strengths in a large number of participants and the utilization of registry-based data on school-related functioning. A cross-sectional design is employed in this study using data from a large population-based sample of adolescents, youth@hordaland, in a linkage to official school registry data, and the current study presents data from *N* = 7,874. The main independent variables were alcohol use and drug use, as well as potential alcohol- and drug-related problems. The dependent variables were registry-based school attendance and grades. All the alcohol- and drug measures included were consistently associated with low GPA (Odds ratios (OR) ranging 1.82–2.21, all *p* < 0.001) and high levels of missed days from school (ORs ranging 1.79–3.04, all *p* < 0.001) and high levels of hours missed from school (ORs ranging 2.17–3.44, all *p* < 0.001). Even after adjusting for gender, age, socioeconomic status and mental health problems all the associations between alcohol and illicit drug use and the school-related outcomes remained statistically significant. Increasing number of indications on alcohol/drug-related problems and increasing levels of alcohol consumption were associated with more negative school-related outcomes. The results suggest that alcohol- and drug use, and particularly alcohol/drug-related problems, are important factors for school-related problems independently of mental health problems.

## Introduction

Adolescents using alcohol and illicit drugs are at risk for prolonged alcohol/drug-related problems (Ellickson et al., 2003), and co-occurrence with mental health problems are often observed among adolescents with alcohol/drug-related problems (Bukstein et al., 1989; Clark et al., 1997). Not least, both alcohol and illicit drug use during adolescence have been found to be associated with long-term negative school-related outcomes, such as lower high school graduation rates (Chatterji, 2006; Renna, 2007; Horwood et al., 2010; Kelly et al., 2015), lower post-secondary educational credentials (Staff et al., 2008), and higher drop-out rates from school (Van Ours and Williams, 2009; Leach and Butterworth, 2012; Brière et al., 2014).

More immediate consequences of alcohol and illicit drug use on school-related problems, such as poor grade achievement and high absence from school, are also highlighted in the scientific literature. Poor grade achievement has been found to be a potent predictor for dropout from school (Janosz et al., 1997), while lower attendance may be an indicator of disengagement from school and is associated with increased substance use (Chou et al., 2006; Henry and Thornberry, 2010). A study by Perini and Marti (2011) found that substance use had no direct effect on drop-out, but had an indirect impact through the intermediate outcomes of poor grades and high school-absence. In other words, short-term school-related problems appear to be important mediators between alcohol/drug use and long-term negative school-related outcomes. Hence, the investigation of how alcohol/drug-related problems are associated with poor grades and high school-absence may be an important step toward a better understanding of adolescents at risk for more long-term negative school-related outcomes.

Some previous studies report that alcohol and illicit drug use is associated with both poorer grades and lower school attendance. For example, adolescent alcohol and illicit drug use are demonstrated to be related to lower self-reported attendance rates (Roebuck et al., 2004; Chou et al., 2006; King et al., 2006; Henry and Thornberry, 2010; Hemphill et al., 2014) and lower self-reported grade achievement (Williams et al., 2003; DeSimone, 2010; Homel et al., 2014; Stiby et al., 2015), while other contributions report weak or non-significant associations between alcohol use and self-reported grades (Sabia, 2010; Brière et al., 2014) and registry-based grades (Balsa et al., 2011). In sum, the literature is not conclusive to whether alcohol and illicit drug use should be regarded as important factors for poor grade achievement and high school-absence or not.

A range of factors should be noted as potential limitations in the previous literature. First, the extent to which alcohol/drug use is associated with negative school-related outcomes may be influenced by the conceptualization of alcohol/drug use. Alcohol use is very prevalent among adolescents (e.g., Windle, 2003), while only a minority of the adolescent drinkers develop more adverse alcohol/drug-related problems (e.g., Olsson et al., 2016). Nevertheless, most previous studies have used single measures of alcohol or drug use—such as either binge drinking, high-level alcohol consumption, heavy drinking, or illicit drug use—and have not attempted to account for how combinations of potential problematic alcohol/drug-related behaviors relate to school-related problems. In our study we employ combined indicators of potential alcohol/drug-related problems, enabling us to evaluate how high-risk alcohol/drug use patterns are associated with poor grades and low school attendance.

Second, previous studies on associations between alcohol and illicit drug use and grade achievement and school attendance have with only a few exceptions (e.g., Hishinuma et al., 2006; Balsa et al., 2011) relied on self-reported measures of school functioning. A study by Balsa et al. (2011) demonstrated that self-reported grades among adolescents with a present alcohol consumption are not only subject to bias, but also that the bias differs by gender. Specifically, boys are more likely to report deflated grades, while girls are more likely to report inflated grades. Therefore, studies employing registry-based information are needed in the investigation on how adolescent alcohol and illicit drug is associated with school functioning. In our study we utilize a linkage with registry-based data on school grades and attendance, which is rare in previous literature.

Third, it is noted that associations between alcohol and drug use and poor school performance may have significant interactions with socioeconomic status (SES), gender and mental health problems (Busch et al., 2014). In particular, mental health problems are demonstrated as influential factors in relation to both adolescent alcohol and illicit drug use (e.g., Chassin et al., 2013) and to negative school-related outcomes (e.g., Lee et al., 2009), and appears to be particularly important factors to take into account when exploring associations between alcohol and illicit drug use and school-related problems. However, very few studies have included mental health problems in the analyses of associations between alcohol and illicit drug use and grade achievement and school attendance (DeSimone, 2010; Stiby et al., 2015). The present study expands on this by including both internalizing symptoms such as anxiety and depression, along with externalizing symptoms such as inattention/hyperactivity and conduct problems as potential confounders. This enables us to investigate whether or not associations between alcohol and drug use and school grades and attendance are also present when mental health problems are accounted for, or if observed associations between alcohol/drug use and school functioning should merely be regarded as an expression of influences from internalizing and/or externalizing traits (e.g., Chassin et al., 2013).

Fourth, some previous studies have demonstrated that alcohol/drug use is associated with general reductions in grade achievement and school attendance (e.g., Roebuck et al., 2004; Balsa et al., 2011). However, the effect sizes are often small, and it may be difficult to interpret whether or not such reductions in school-related functioning should be regarded as indicators of school-related problems. In our study we address this “interpretation” issue, by investigating associations between alcohol/drug use and school-related problems, defined as low-levels of grade achievement and high-levels of school absence. In this respect, our study provides new knowledge with regard to how alcohol/drug use is associated with short-term school-related problems.

In sum, the present study contributes to the understanding of the association between adolescent alcohol- and illicit drug use and academic achievement in terms of grades and attendance rates. Utilizing a unique linkage between a large scale Norwegian population-based study among adolescents and official school-registry data on student's grades and attendance rates, we aimed to investigate the cross-sectional association between alcohol- and illicit drug use, and alcohol/drug-related problems, and negative school-related outcomes, including low GPA and high number of days and hours missed from class. Importantly, we use official registry based data on both grades and attendance rates, thereby obviating self-report bias in relation to the school-related outcomes. Additionally, we employed a range of indicators for both alcohol and illicit drug use, along with potential alcohol and drug-related problems, thereby enabling us to investigate associations with school-related functioning across different patterns of alcohol and illicit drug use.

## Materials and methods

### Study population

We employed data from the youth@hordaland study, which aimed at providing data on child and adolescent mental health, lifestyle, school performance and use of health services. All adolescents born between 1993 and 1995 living in Hordaland county in western Norway were invited to participate (*N* = 19,430), and of these 10,257 adolescents chose to participate, giving a participation rate of 53%. After deletion of participants not giving consent to use data from the school registry (*N* = 682), and those having missing information on either school registry data (*N* = 1,190) or alcohol- and illicit drug use (*N* = 511), the final number of participants was 7,874. 52% of the participants were girls, and the mean age in the sample was 17.4 (standard deviation 0.8).

Youth@hordaland is a cross-sectional population-based study carried out during early 2012, and data was collected from adolescents in upper secondary school. The adolescents received information per email and one school hour was used to complete the questionnaires at school. In addition, adolescents not going to school received the questionnaires by mail at their home address, and also mental health services and other institutions were contacted to let adolescents from these settings participate. The questionnaires used in the youth@hordaland study were web-based, and electronic informed consent was obtained from all participants. The study was approved by the Regional Committee for Medical and Health Research Ethics in Western Norway.

In order to provide access to medical care, easy accessible information on mental health services was made available for the adolescents who participated in the youth@hordaland study. Additionally, a direct phone number to the research staff was provided, by which they could call to receive more information. Also, personnel within school health services were informed about the survey, and therefore enabled to be present for the adolescents by the time they answered the questionnaire.

A previous population-based study found that the geographical area from where the adolescents came, Hordaland county, to be regarded as representative of the general Norwegian population (Folkehelseinstituttet, 2010).

### Exposure: alcohol- and illicit drug use

Self-reported measures of alcohol- and illicit drug use were our main independent variables.

#### Ever tried alcohol

Based on a single item “Have you ever tried alcohol?,” a dichotomous variable was constructed (Yes/No). *N* = 6,159 (78.3%) of the sample reported to having consumed alcohol.

#### Ever tried illicit drugs

Another dichotomous variable was constructed based on a single item “Have you ever tried hash, marijuana or other narcotic substances?” (Yes/No). *N* = 788 (10.0%) of the sample reported to having tried illicit drugs.

#### High-level alcohol consumption

Items measuring self-reported glasses of beer, cider, wine, spirits and illegally distilled spirits usually consumed during 14 days were added up. A total of *N* = 4,503 (61.2%) of the sample reported a present alcohol consumption. The high-level alcohol consumption variable was defined as the above 90th gender-specific percentile alcohol consumption among the adolescents with a present alcohol consumption, and a dichotomous variable was created for high-level alcohol consumption (*N* = 453). In addition, based on the continuous distribution of alcohol consumption in the sample an ordinal gender-specific variable of alcohol consumption was constructed, including seven levels from never used alcohol to consumption above 90th centile.

#### Frequent alcohol intoxication

Frequency of intoxication was measured based on the question: “Have you ever consumed so much alcohol that you were clearly intoxicated (drunk)?” The original item had five categories ranging from “No, never” to “Yes, more than 10 times.” Frequent intoxication was defined as drinking so much that one was clearly intoxicated more than 10 times (Skogen et al., 2014), and on this basis a dichotomous variable was created. *N* = 1,588 (20.2%) of the sample reported frequent intoxication.

#### Positive crafft score

Alcohol and drug-related problems were measured using the six-item, validated scale CRAFFT. This scale has been designed to identify possible alcohol-and drug related problems among adolescents, and has been demonstrated to have acceptable sensitivity and specificity at a cut-off of ≥2 (Dhalla et al., 2011). A dichotomous variable separating those above the cut-off of ≥2 on CRAFFT from those below the cut-off were calculated. *N* = 1,664 (21.2%) of the sample scored above the CRAFFT cut-off, and were operationalized to indicate potential alcohol- or illicit drug-related problems. In our sample the Cronbach's α of the CRAFFT scale was 0.67.

#### Any and total potential alcohol/drug-related problems

We constructed a dichotomous measure for any potential alcohol/drug-related problems, indicating whether or not an adolescent had a positive score for either having frequent alcohol intoxication, high-level alcohol consumption, a positive CRAFFT-score or having tried illicit drugs. *N* = 2,710 (34.4%) of the sample had any potential alcohol/drug-related problem. Similarly, we constructed an ordinal variable for total potential alcohol/drug-related problems, in which we summed up the number of positive scores on frequent alcohol intoxication, high-level alcohol consumption, a positive CRAFFT-score or having tried illicit drugs. A total of 5,164 (66.1%) had none, 1,439 (18.4%) had one, 743 (9.5%) had two, 384 (4.9%) had three, and 84 (1.1%) had four of these potential alcohol/drug-related problems.

### Outcome: registry-based information about school performance and attendance

Academic grades were provided by official school registry in Hordaland County. In Norway, secondary schools use a scale running from 1 to 6, with 6 being the highest grade (outstanding competence), 2 being the lowest passing grade (low level of competence), and a 1 is a “fail” (no qualified competence). The grade point average (GPA) was calculated as the average of the student's grades during their time at the school. Mean combined GPA in the sample was 3.85 (standard deviation 0.80). Based on the continuous distribution of GPA in the sample, we dichotomized GPA under/above the 10th gender-specific percentile, constructing a variable indicating low GPA for adolescents scoring below this threshold. 859 (10.9%) of the adolescents had a low GPA.

Official registry-based data on attendance rates were also provided by official registry data from the Hordaland County, and they included both days and school hours of absence for the last semester (6 months). The mean number of days missed in the sample was 4.02 (standard deviation 5.04), while the mean number of hours missed was 7.51 (standard deviation 11.10). Based on the continuous distribution in the sample of respectively days and hours missed from school, we constructed two variables indicating high number of days and high number of hours missed from school for adolescents which were dichotomized under/above the 90th gender-specific levels of respectively number of days and hours they did not attend school. 721 (9.2%) of the adolescents had a high number of days missed, and 767 (9.7%) had a high number of hours missed from school.

### Included co-variates

Demographic information and self-reported symptoms of depression, anxiety, inattention and hyperactivity (ADHD), and conduct problems were included and used as control variables in the main regression analyses.

#### Demographic information

Age and gender were retrieved from registry data. In addition, socioeconomic status (SES) was collected by a self-reported item of perceived family economy as either (1) “about the same as others” (67%) (2) “better than others” (26%), or (3) “worse than others” (7%). Information on maternal and paternal educational attainment was collected by two self-report items separating the parental educational attainment variable into only primary school, high school, or more than 4 years of University or higher education. Both perceived family economy and parental educational attainment have been used as measures for SES in previous publications (e.g., Skogen et al., 2014) and have been found to be comparably associated with mental health problems (Bøe et al., 2012). The variables of perceived family economy, paternal educational attainment, and maternal educational attainment were all used as a measure of socioeconomic status (SES), and were included as control variables in the logistic regression models for the associations between alcohol and illicit drug use, and potential alcohol/drug-related problems, and the school-related outcomes of interest.

#### Mental health problems

Symptoms of depression was assessed using the short version of the Mood and Feelings Questionnaire (SMFQ) (Thapar and McGuffin, 1998). The SMFQ consist of 13 items assessing depressive symptoms rated on a 3-point scale, ranging from “Not true,” “Sometimes true,” and “True.” A continuous measure of the SMFQ has recently been validated among Norwegian adolescents (Lundervold et al., 2013), and was used in the regression analyses in our study. In our sample the Cronbach's α of the SMFQ was 0.88.

Symptoms of anxiety were correspondingly identified by employing the five-item inventory SCARED, which is a short form of the 41-item full version screening inventory for anxiety disorders (Birmaher et al., 1999). A continuous measure of the SCARED was used in our regression analyses. The Cronbach's α of the short form of the SCARED instrument in our sample was 0.69.

Symptoms of inattention and hyperactivity were measured using an official Norwegian translation of the Adult ADHD Self-report Scale (ASRS) (Kessler et al., 2007). The ASRS instrument is an 18-item self-report scale, where 9 items construct the hyperactivity-impulsivity subscale and the 9 other items construct the inattention subscale. Responses are given on a 5-point scale ranging from “Never” to “Very often.” The Cronbach's α of the ASRS in our sample was 0.89.

Symptoms of conduct problems were measured using the Youth Conduct Disorder (YCD) instrument, consisting of 8 items which are part of the Diagnostic Interview Schedule for Children Predictive Scales (DPS) (Lucas et al., 2001). The DPS scale has been shown to accurately determine adolescents who are at high probability of meeting diagnostic criteria for conduct disorder. The Cronbach's α of the YCD in our sample was 0.79.

### Statistical analysis

The following statistical analyses were conducted: First, the sample was described according to age, gender, socioeconomic status, school-related functioning, and alcohol and drug use (Table 1). Second, odds ratios of the associations between alcohol/drug-related variables and the school-related variables were computed using logistic regression models (Table 2). More specifically, crude regression models were utilized, followed by adjustments for age, gender and SES, and finally adjusted for age, gender, SES, and mental health problems. Third, logistic regression analyses were conducted for the associations between ordinal number of indications on alcohol/drug-related problems and school-related outcomes, and also these associations were adjusted for the potential confounding by age, gender, SES and mental health problems. Fourth, crude and adjusted logistic regression models were conducted for associations between ordinal levels of alcohol consumption and the school-related outcomes. All analyses were performed using STATA V.14.0 (StataCorp., 2015).

**Table 1:** Demographical and mental health-related characteristics in the adolescents of the sample (*n* = 7,874).

**Demographics**	**Girls**	**Boys**	***p*-value**
Girls, %	52.3		<0.001
Age, mean (SD)	17.4 (0.8)	17.4 (0.8)	n.s.
Perceived family economy, %			<0.01
Below average	8.1	6.2	
Average	70.3	64.6	
Above average	21.6	29.3	
Mothers education, %^a^			n.s.
University/college	11.4	9.7	
High school	40.9	43.3	
Primary school	47.7	47.0	
Fathers education, %^b^			n.s.
University/college	11.5	10.8	
High school	46.3	48.1	
Primary school	42.3	41.1	
**ALCOHOL AND ILLICIT DRUG USE**			
Tried alcohol, %	81.1	75.2	<0.001
Tried illicit drugs, %	8.5	11.7	<0.001
CRAFFT-score ≥ 2, %	22.9	19.4	<0.001
Frequent drinking to intoxication, %	20.0	20.3	n.s.
**MENTAL HEALTH PROBLEMS**^c^			
Depression symptoms, mean (SD)	7.29 (6.04)	4.08 (4.87)	<0.001
Anxiety symptoms, mean (SD)	2.02 (1.91)	0.93 (1.51)	<0.001
ADHD symptoms, mean (SD)	28.32 (10.07)	25.08 (10.85)	<0.001
Conduct problems symptoms, mean (SD)	0.38 (0.95)	0.71 (1.48)	<0.001
**SCHOOL-RELATED FUNCTIONING**			
GPA, mean (SD)	3.95 (0.78)	3.73 (0.80)	<0.001
Days missed, mean (SD)	4.51 (5.26)	3.49 (4.72)	<0.001
Hours missed, mean (SD)	7.58 (10.68)	7.44 (11.55)	n.s.

*CRAFFT: screening scale for identification of potential problematic alcohol and drug use among adolescents*.

a *Only includes those who with valid response on mothers education (n = 5,937), excluding those having answered that they don't know (n = 1,881)*.

b *Only includes those who with valid response on fathers education (n = 5,819), excluding those having answered that they don't know (n = 1,979)*.

c *The measure for mental health problems includes depression (SMFQ), anxiety (SCARED), inattention/hyperactivity (ASRS), and conduct problems (YCD)*.

**Table 2:** Logistic regression analyses of associations between alcohol- and illicit drug use and negative school-related outcomes.

	**Low GPA**		**High number of days missed from school**		**High number of hours missed from school**	
	**OR**	**95% CI**	**OR**	**95% CI**	**OR**	**95% CI**
**TRIED ALCOHOL (*****n*** = **6,159)**						
Crude	1.98^***^	1.70, 2.31	2.20^***^	1.74, 2.78	2.99^***^	2.32, 3.84
Adjusted for age, gender and SES	2.12^***^	1.79, 2.50	1.85^***^	1.44, 2.36	2.59^***^	1.99, 3.36
+ adj for mental health problems^a^	1.83^***^	1.54, 2.18	1.60^***^	1.25, 2.06	2.09^***^	1.60, 2.73
**TRIED ILLICIT DRUGS (*****n*** = **788)**						
Crude	1.82^***^	1.36, 2.44	3.04^***^	2.51, 3.69	3.44^***^	2.86, 4.14
Adjusted for age, gender and SES	1.92^***^	1.42, 2.58	2.81^***^	2.29, 3.44	3.12^***^	2.57, 3.79
+ adj for mental health problems^a^	1.48^*^	1.09, 2.01	2.31^***^	1.87, 2.86	2.28^***^	1.86, 2.80
**POSITIVE CRAFFT-SCORE (*****n*** = **1,664)**						
Crude	2.04^***^	1.65, 2.52	2.41^***^	2.04, 2.83	2.41^***^	2.06, 2.83
Adjusted for age, gender and SES	2.05^***^	1.65, 2.54	2.13^***^	1.80, 2.52	2.21^***^	1.88, 2.60
+ adj for mental health problems^a^	1.60^***^	1.27, 2.00	1.70^***^	1.42, 2.04	1.53^***^	1.28, 1.83
**FREQUENT ALC INTOXICATION**^b^ **(*****n*** = **1,588)**						
Crude	2.14^***^	1.68, 2.72	1.79^***^	1.51, 2.14	2.17^***^	1.84, 2.56
Adjusted for age, gender and SES	2.31^***^	1.80, 2.96	1.63^***^	1.35, 1.96	2.05^***^	1.72, 2.44
+ adj for mental health problems^a^	2.04^***^	1.59, 2.63	1.44^***^	1.19, 1.74	1.70^***^	1.42, 2.04
**HIGH ALCOHOL CONSUMPTION**^c^ **(*****n*** = **453)**						
Crude	2.12^***^	1.42, 3.17	1.97^***^	1.51, 2.56	2.68^***^	2.11, 3.40
Adjusted for age, gender and SES	2.00^**^	1.33, 3.00	1.71^***^	1.29, 2.26	2.51^***^	1.96, 3.23
+ adj for mental health problems^a^	1.66^*^	1.10, 2.51	1.48^**^	1.11, 1.97	1.90^***^	1.46, 2.47
**ANY ALCOHOL/DRUG PROBLEM (*****n*** = **2,710)**						
Crude	2.21^***^	1.86, 2.63	2.49^***^	2.13, 2.90	2.82^***^	2.42, 3.28
Adjusted for age, gender and SES	2.30^***^	1.92, 2.76	2.17^***^	1.85, 2.56	2.60^***^	2.21, 3.04
+ adj for mental health problems^a^	1.90^***^	1.58, 2.30	1.85^***^	1.56, 2.19	1.98^***^	1.67, 2.35

*N = 7,874 (girls n = 4,121, boys, n = 3,753)*.

a *The measure for mental health problems includes depression (SMFQ), anxiety (SCARED), inattention/hyperactivity (ASRS), and conduct problems (YCD)*.

b *Drinking alcohol to intoxication more than 10 times*.

c *≥90th percentile gender-specific alcohol consumption (n = 453) among adolescents with a present alcohol consumption (n = 4,503)*.

Bold font denotes statistical significant mean differences at

*** *p < 0.001*,

** *p < 0.01*,

* *p < 0.05*.

## Results

### Demographical and mental health-related characteristics in the sample

The adolescents which were excluded (*n* = 2,383) due to either non-consent for the usage of school registry data, or to missing information on either school registry data or alcohol- and illicit drug use, were found to deviate slightly from the adolescents of the final sample. They were more likely to be younger (mean difference −0.13, *p* < 0.001), to have mothers with higher educational attainment (mean difference 0.11, *p* < 0.01) and fathers with higher educational attainment (mean difference 0.15, *p* < 0.001), to have more symptoms of depression measured by SMFQ (mean difference 0.76, *p* < 0.001), and to have more symptoms of inattention or hyperactivity measured by ASRS (mean difference 0.74, *p* < 0.01). The adolescents who were excluded from the sample and which had valid responses on alcohol and illicit drug use, were found to be less likely to have tried alcohol than the adolescents in the included sample (73.1% compared to 78.3%, *p* < 0.001), but did not deviate on having tried illicit drugs or on the extent to which they had a positive CRAFFT score.

The final sample consisted of *N* = 7,874 participants. Table 1 outlines the main demographical characteristics of the final sample, as well as the characteristics on alcohol and illicit drugs and school-related variables. The mean age of the sample was 17.4 years (standard deviation 0.83), and the sample included more girls (52.3%, *p* < 0.001). Regarding alcohol- and illicit drug use, a total of 78.3% of the sample had used alcohol, 10.0% had tried illicit drugs, 21.2% scored above the CRAFFT cut-off at ≥2, indicating a problematic alcohol and drug use, and 20.2% of the sample reported to having been intoxicated by alcohol more than 10 times.

Some gender differences were found in the sample. Lower perceived family economy were more common among girls (*p* < 0.01). Girls had higher mean scores compared with boys on symptoms of depression (7.29 vs. 4.08, *p* < 0.001), anxiety (2.02 vs. 0.93, *p* < 0.001) and ADHD (28.32 vs. 25.08, *p* < 0.001), while boys had higher mean scores compared with girls on symptoms of conduct problems (0.71 vs. 0.38, *p* < 0.001). Girls were also more likely to having ever tried alcohol (81.1 vs. 75.2%, *p* < 0.001) and to have a positive CRAFFT score (22.9 vs. 19.4%, *p* < 0.001), while boys were more likely to having tried illicit drugs (11.7 vs. 8.5%, *p* < 0.001). Finally, girls had a higher mean GPA (3.95 vs. 3.74, *p* < 0.001) and a higher number of days missed from school (4.51 vs. 3.49, *p* < 0.001) compared to boys.

### Alcohol- and illicit drug use and school-related outcomes

Table 2 depicts the crude and adjusted associations between alcohol- and illicit drug use and the school-related outcomes of GPA, days missed from school, and hours missed from school. As detailed in this table, all the alcohol- and drug measures in the crude model were consistently associated (all *p* < 0.001) with low GPA (Odds ratios (OR) ranging 1.82–2.21) and high number of days missed (ORs ranging 1.79–3.04) and hours missed (ORs ranging 2.17–3.44).

When adjusting for age, gender, self-reported family SES and mental health problems the estimated associations were somewhat altered, but even in the fully adjusted model, all measures of alcohol- and illicit drug use still showed statistically significant associations with low GPA (Adjusted odds ratios (AOR) ranging from 1.48 to 2.04, all *p* < 0.05), and high number of days missed (AORs ranging 1.44–2.31, all *p* < 0.01) and high number of hours missed (AORs ranging from 1.53 to 2.28, all *p* < 0.001).

### Ordinal levels of potential alcohol/drug-related problems and school-related outcomes

Table 3 outlines the associations between ordinal number of indications on alcohol/drug-related problems and school-related outcomes. For GPA the odds ratios ranged from 2.01 to 2.91 (all *p* < 0.001) in crude models, and from 1.78 to 2.35 in fully adjusted models (all *p* < 0.01). For days missed from school the odds ratios ranged from 2.08 to 4.69 in crude models and from 1.72 to 3.13 in fully adjusted models (all *p* < 0.001), while the odds ratios for hours missed from school ranged from 2.02 to 5.17 in crude models and from 1.62 to 2.93 in fully adjusted models (all *p* < 0.001). In both the crude and adjusted models there were statistically significant monotonous trends in the associations between increasing levels of potential alcohol/drug-related problems and increasingly adverse school-related outcomes (all *p* < 0.001), indicating that more indicators of alcohol/drug-related problems were associated with more negative school-related outcomes.

**Table 3:** Logistic regression analyses of associations between ordinal levels of potential alcohol/drug-related problems and negative school-related outcomes.

	**Low GPA**^a^		**High number of days missed from school**^a^		**High number of hours missed from school**^a^	
	**OR**	**95% CI**	**OR**	**95% CI**	**OR**	**95% CI**
No alc/drug problems (*n* = 5,164)	(Base)		(Base)		(Base)	
**1 INDICATION OF ALC/DRUG PROBL (*****n*** = **1,439)**						
Crude	2.01^***^	1.62, 2.49	2.08^***^	1.71, 2.53	2.02^***^	1.66, 2.47
Adjusted for age, gender and SES	2.03^***^	1.63, 2.53	1.90^***^	1.55, 2.33	1.92^***^	1.57, 2.35
+ adj for mental health problems^b^	1.78^***^	1.42, 2.23	1.72^***^	1.39, 2.12	1.63^***^	1.32, 2.01
**2 INDICATIONS OF ALC/DRUG PROBL (*****n*** = **743)**						
Crude	2.81^***^	2.02, 3.90	2.40^***^	1.89, 3.06	3.13^***^	2.50, 3.91
Adjusted for age, gender and SES	2.92^***^	2.08, 4.09	2.13^***^	1.66, 2.73	2.85^***^	2.26, 3.59
+ adj for mental health problems^b^	2.35^***^	1.66, 3.31	1.80^***^	1.39, 2.33	2.17^***^	1.70, 2.76
**3–4 INDICATIONS OF ALC/DRUG PROBL (*****n*** = **468)**						
Crude	2.91^***^	1.84, 4.60	4.69^***^	3.61, 6.11	5.17^***^	4.00, 6.69
Adjusted for age, gender and SES	2.98^***^	1.87, 4.74	3.95^***^	2.98, 5.22	4.36^***^	3.32, 5.71
+ adj for mental health problems^b^	2.17^**^	1.35, 3.48	3.13^***^	2.33, 4.22	2.93^***^	2.17, 3.91

*N = 7,874 (girls n = 4,121, boys, n = 3,753)*.

a *p-value for trend in the association between potential alcohol/drug-related problems and school-related outcomes, all p < 0.001*.

b *The measure for mental health problems includes depression (SMFQ), anxiety (SCARED), inattention/hyperactivity (ASRS), and conduct problems (YCD)*.

Bold fonts denotes statistically significant associations:

*** *p < 0.001*,

** *p < 0.01*.

### Ordinal levels of alcohol consumption and school-related outcomes

Table 4 depicts the crude associations between ordinal levels of alcohol consumption and the school-related outcomes of low GPA, high number of days missed from school, and high number of hours missed from school. As detailed in the table, increasing levels of alcohol consumption were associated with lower GPA and a higher number of days and hours missed from school, and for all the school-related outcomes of interest these monotonous trends were statistically significant in both the crude and fully adjusted models (all *p* < 0.001).

**Table 4:** Logistic regression analyses of associations between ordinal levels of alcohol consumption^a^ and negative school-related outcomes.

	**Low GPA**^b^		**High number of days missed from school**^b^		**High number of hours missed from school**^b^	
	**OR**	**95% CI**	**OR**	**95% CI**	**OR**	**95% CI**
**CRUDE MODEL**						
Never consumed alcohol	(Base)		(Base)		(Base)	
Non-consumption	1.42^**^	1.14, 1.76	1.61^**^	1.19, 2.19	1.74^**^	1.25, 2.42
0.1–19.9th percentile	1.21	0.96, 1.52	1.59^**^	1.14, 2.21	1.88^***^	1.32, 2.67
20–49.9th percentile	1.99^***^	1.59, 2.48	1.97^***^	1.49, 2.62	2.64^***^	1.97, 3.55
50–79.9th percentile	3.24^***^	2.50, 4.21	2.77^***^	2.12, 3.64	4.03^***^	3.04, 5.34
80–89.9th percentile	3.28^***^	2.15, 5.01	3.77^***^	2.70, 5.27	5.09^***^	3.61, 7.18
90–100th percentile	2.97^***^	2.01, 4.40	3.05^***^	2.17, 4.29	6.05^***^	4.37, 8.40
**FULLY ADJUSTED**^c^						
Never consumed alcohol	(Base)		(Base)		(Base)	
Non-consumption	1.35^**^	1.08, 1.69	1.36	0.99, 1.88	1.49^*^	1.06, 2.10
0.1–19.9th percentile	1.34^*^	1.05, 1.72	1.30	0.91, 1.84	1.60^*^	1.12, 2.30
20–49.9th percentile	2.01^***^	1.58, 2.56	1.50^**^	1.11, 2.03	1.98^***^	1.45, 2.72
50–79.9th percentile	3.14^***^	2.37, 4.17	1.83^***^	1.36, 2.46	2.83^***^	2.09, 3.83
80–89.9th percentile	2.82^***^	1.82, 4.38	2.50^***^	1.74, 3.59	3.50^***^	2.43, 5.04
90–100th percentile	2.62^***^	1.75, 3.94	2.06^***^	1.42, 2.98	4.01^***^	2.81, 5.71

*N = 7,874 (girls n = 4,121, boys, n = 3,753)*.

a *Presented alcohol level consumption percentiles are calculated among those adolescents who report to have an actual alcohol consumption*.

b *p-value for trend in the association between alcohol variable and school-related variable: all p < 0.001*.

c *Adjusted for the confounding of age, gender, SES and mental health problems*.

Bold fonts denotes statistically significant associations:

*** *p < 0.001*,

** *p < 0.01*,

* *p < 0.05*.

## Discussion

### Main findings

The aim of this study was to investigate the associations between alcohol and drug use, and alcohol/drug-related problems, and school-related problems measured by low GPA and high number of days and hours missed from school. In short, all the alcohol- and drug measures included were consistently associated with low GPA and high number of days and hours missed from school. In this respect, our study supports several previous studies which have reported that adolescent alcohol- and illicit drug use are associated with lower academic achievement (e.g., Williams et al., 2003; DeSimone, 2010; Homel et al., 2014) and increased absence from school (e.g., Roebuck et al., 2004; Hemphill et al., 2014), while it contradicts some recent studies which indicates that alcohol/drug use should not be regarded as particularly important factors for school-related functioning (Sabia, 2010; Balsa et al., 2011; Brière et al., 2014).

Few previous studies have investigated the extent to which the associations between alcohol- and illicit drug use/problems and negative school-related outcomes may be confounded by mental health problems, along with SES, gender and age. Theoretically, this is a highly relevant issue, as the association between school-related adverse outcomes and adolescent alcohol and illicit drug is likely to be complex and not necessarily causal in its nature (e.g., Busch et al., 2014; Stiby et al., 2015). It is suggested that the often observed association between alcohol and illicit drug use and school-related outcomes may be either direct (e.g., Latvala et al., 2014), that it may be a reverse association (e.g., Crosnoe, 2006; Brière et al., 2014), or that it may be caused by third factors which operate in ways that creates the observed association (e.g., Crosnoe, 2006). Importantly, as developmental models conceptualize alcohol and illicit drug use among adolescents as expressions of a broader tendency toward either internalizing problems or externalizing problems (e.g., Chassin et al., 2013), observed associations between alcohol and illicit drug use and school-related outcomes may therefore be hypothesized to merely be a marker of these broader tendencies.

In our study we adjusted the associations between alcohol- and illicit drug use/problems for the potential confounding from gender, age, socioeconomic factors and mental health problems, in accordance with recommendations from previous studies on this topic (e.g., Sabia, 2010; Balsa et al., 2011). We found that these confounders accounted for some, but not all of the association. In the fully adjusted models all associations between alcohol and illicit drug use/problems and the negative school-related outcomes were still statistically significant, although the size of the odds ratios were generally reduced, particularly when mental health problems were entered into the model. Therefore, our findings suggest that alcohol- and illicit drug use, and potential alcohol/drug-related problems, has a unique contribution to the association with negative school-related outcomes, which only in part may be attributed to the presence of mental health problems, and therefore to the broader tendencies to either internalizing or externalizing problems.

These findings extend the existing literature. A previous study by Sabia (2010) reported that after adjusting for psychological well-being and factors and individual changes in alcohol use, much of the association between alcohol use and grades disappeared. Similarly, Hemphill et al. (2014) reported that most of the association between alcohol use and subsequent grades and school attendance disappeared when adjusting for a range of individual, family, peer and school-related confounders. In our study the association between alcohol and illicit drug use and school-related outcomes consistently remained robust and statistically significant after adjusting for age, gender, socioeconomic status, and mental health.

We also wanted to explore how potential alcohol/drug-related problems contributed to the association between alcohol/drug use and negative school-related outcomes. The CRAFFT instrument is a widely used screening tool for potential alcohol/drug-related problems among adolescents, providing a broader perspective of adolescent alcohol and illicit drug use than self-reported frequency of alcohol and illicit drug use alone (Agley et al., 2015). CRAFFT has been found to correlate with other measures of substance use in adolescents, supporting its efficacy as a screening tool among adolescents (Pilowsky and Wu, 2013; Skogen et al., 2013; Oesterle et al., 2015). In the present findings potential alcohol/drug-related problems as measured by the CRAFFT instrument were consistently associated with negative school-related function in terms of low GPA and high number of days and hours missed from school. The magnitude of the associations between alcohol/drug-related problems as indicated by a positive CRAFFT score and school-related problems was comparable to the magnitudes of the associations between the other included measures of alcohol/drug use and school-related problems. However, we also added supplementary measures for potential alcohol/drug-related problems, in terms of ordinal number of indicators on problematic alcohol and illicit drug use, and we found that higher number of indicators on potential alcohol/drug-related problems was associated with higher levels of school-related problems.

Similarly, the associations with negative school-related outcomes increased with ordinal increases of alcohol consumption levels. This tendency was found with regards to all the negative school-related measures. To our knowledge no previous studies have investigated how increasing levels of either potential alcohol/drug-related problems or alcohol consumption correspond to negative school-related outcomes, such as GPA or school attendance. A previous study reported a dose-response effect between cannabis use and results on standardized assessment test at age 16 (Stiby et al., 2015), while we have not found other studies reporting on how ordinal or continuous levels of alcohol- and illicit drug use/problems are associated with either school grades or school attendance rates. In short, our findings indicate that increasing levels of indicators of alcohol/drug-related problems and increasing levels of alcohol consumption are associated with increasing school-related problems, indicating that high-risk alcohol/drug use is strongly associated with school-related problems. We did not have available data to investigate if these patterns also applied to increasing levels of illicit drug use; something which should be addressed in future studies.

A final noteworthy finding in our study was that the association between alcohol use and negative school-related outcomes were not constricted to only a certain type of drinking pattern, and the magnitude on the association with the school-related problems only slightly varied across different measures of alcohol use. A previous study reported that binge drinking, but not alcohol use without binging, were associated with somewhat lower GPA (DeSimone, 2010). Although we did not have a variable which directly measured binge drinking, we found that both frequent alcohol intoxication and other measures of alcohol use were consistently associated with lower grade achievement, thereby contradicting the findings from DeSimone and colleges. Overall, our findings suggest that all types of alcohol and illicit drug use were associated with negative school-related outcomes, with comparable magnitudes between all measures of alcohol/drug use, and that increasing numbers of indicators for potential alcohol/drug-related problems was associated with more school-related problems.

### Implications

Our study suggest that alcohol and illicit drug use should be regarded as important factors for school-related functioning among adolescents, and that alcohol and illicit drug use has a unique contribution to negative school-related outcomes in terms of low GPA and high number of days and hours missed from school. Although positive associations were found for all included measures of alcohol/drug use, the most high-risk alcohol/drug use patterns had clearly the strongest associations with school-related problems. An important implication of this study is that alcohol/drug use, and particularly the most risky patterns of alcohol/drug use, should be targeted in initiatives aiming at better school-functioning among adolescents. Future studies should be encouraged to investigate to what extent short-term school problems, such as poor grades and high school-absence, serve as mediators between alcohol/drug use and more long-term negative school-related outcomes, as very few studies have explored this possibility (Perini and Marti, 2011).

### Strengths and limitations

The present study has several strengths. First, the sample consists of a well-defined population-based sample of adolescents in the age 16–19 years, which is sufficiently large to enable a detailed investigation of main effects between alcohol- and illicit drug use and school-related outcomes, along with sub-analyses of ordinal levels of alcohol consumption and potential alcohol/drug-related problems. Second, a unique linkage to the official school-registry was utilized, facilitating an investigation of objective data on GPA and days and hours missed from school. Third, the data from our study sample is recent, thus allowing for an updated view into the current status of alcohol- and drug use and its association with school-related outcomes. Fourth, the study used several measures of alcohol- and drug use, including a validated measure of potential alcohol- and drug related problems, i.e., the CRAFFT instrument, along with measures of increasing levels of potential indicators for alcohol/drug-related problems. Fifth, other standardized measures of symptoms on anxiety, depression as well as hyperactivity and inattention, were used in our study. Finally, we adjusted our analyses for a range of potential confounders on the association between alcohol and illicit drug use and school-related problems.

The present study has some limitations. First, the study has a cross-sectional design, and it is therefore not possible to draw conclusion on causality between alcohol and illicit drug use and school-related outcomes based on this study. Second, due to some adolescents not giving consent to use registry data, to missing data in the school-registry, and missing responses on the alcohol- and illicit drug variables, a total of 23% (*n* = 2,383) of the school-attending adolescents aged 16–19 were not included in our study. Our analyses revealed that this excluded group reported somewhat higher education among their parents, they were younger, and had more symptoms from depression and ADHD. Additionally, they were less likely to have tried alcohol. In sum, this may affect the generalizability of our findings among the school-attending adolescents. Third, the questionnaire which measured both the alcohol- and illicit drug use and the mental health variables, were solely based on self-report. This may have led to a bias in the data due to misclassification of the independent and control variables used in this study. The use of self-reported measures does not imply the presence of actual psychiatric or substance-related diagnoses, and the lack of clinical interviews in the collection of data on mental health and alcohol- and drug use adds as a limitation to our study. Fourth, we did not include chronic illness as a confounding variable. We may not rule out that chronic illness may have played a confounding role on the association between alcohol/drug use and school-related problems, something which could be addressed in future studies. Fifth, we did not investigate cumulative effects from alcohol/drug use in combination with other potential risk factors such as mental health problems on school-related problems, as this issue is beyond the scope of the present paper. Finally, residual confounding may be an issue.

## Conclusion

Adolescence is a time period where it is common to experiment with alcohol and illicit drugs, and many of the adolescents which display a risky alcohol and drug use will neither develop long-lasting substance problems nor school- or later work-related problems. However, the results from our study indicate that alcohol- and drug-related problems are important factors in school-related functioning. Importantly, alcohol- and illicit drug use, and potential alcohol/drug-related problems, were consistently associated school-related problems, even when no mental health problems are present, and the associations were particularly strong among adolescents with the most risky alcohol/drug use patterns. Our study highlight the need to keep adolescent's use of alcohol and illicit drugs as an important concern for prevention initiatives at all levels of the society surrounding the adolescents. In particular, efforts aiming to increase school-related functioning among adolescents should be aware of the important role of reducing levels of alcohol and illicit drug use (e.g., Engberg and Morral, 2006). Measures should be made to ensure a proper identification of adolescents at the highest risk for problematic alcohol- and illicit drug use, along with access to and utilization of health care services when needed; while initiatives aiming at reducing total levels of alcohol- and drug use among adolescents are also encouraged.

## Author contributions

OH has carried out the literature review for the introduction and discussion sections, conducted the statistical analyses, and has written the manuscript. MH, JH, and JS has been involved in the preparation and conduction of the statistical analyses, and have reviewed and contributed to all parts of the written manuscript.

### Conflict of interest statement

The authors declare that the research was conducted in the absence of any commercial or financial relationships that could be construed as a potential conflict of interest.

## References

[B1] Agley J. Gassman R. A. Jun M. Nowicke C. Samuel S. (2015). Statewide administration of the CRAFFT screening tool: highlighting the spectrum of substance use. Subst. Use Misuse 50, 1668–1677. 10.3109/10826084.2015.1027930 26579780

[B2] Balsa A. I. Giuliano L. M. French M. T. (2011). The effects of alcohol use on academic achievement in high school. Econ. Educ. Rev. 30, 1–15. 10.1016/j.econedurev.2010.06.015 21278841 PMC3026599

[B3] Birmaher B. Brent D. A. Chiappetta L. Bridge J. Monga S. Baugher M. (1999). Psychometric properties of the screen for child anxiety related emotional disorders (SCARED): a replication study. J. Am. Acad. Child Adolesc. Psychiatry 38, 1230–1236. 10.1097/00004583-199910000-00011 10517055

[B4] Bøe T. Øerland S. Lundervold A. J. Hysing M. (2012). Socioeconomic status and children's mental health: results from the bergen child study. Soc. Psychiatry Psychiatr. Epidemiol. 47, 1557–1566. 10.1007/s00127-011-0462-9 22183690

[B5] Brière F. N. Fallu J.-S. Morizot J. Janosz M. (2014). Adolescent illicit drug use and subsequent academic and psychosocial adjustment: an examination of socially-mediated pathways. Drug Alcohol Depend. 135, 45–51. 10.1016/j.drugalcdep.2013.10.029 24322005

[B6] Bukstein O. G. Brent D. A. Kaminer Y. (1989). Comorbidity of substance abuse and other psychiatric disorders in adolescents. Am. J. Psychiatry 146, 1131–1141. 10.1176/ajp.146.9.1131 2669535

[B7] Busch V. Loyen A. Lodder M. Schrijvers A. J. van Yperen T. A. de Leeuw J. R. (2014). The effects of adolescent health-related behavior on academic performance a systematic review of the longitudinal evidence. Rev. Educ. Res. 84, 245–274. 10.3102/0034654313518441

[B8] Chassin L. Sher K. J. Hussong A. Curran P. (2013). The developmental psychopathology of alcohol use and alcohol disorders: research achievements and future directions. Dev. Psychopathol. 25, 1567–1584. 10.1017/S0954579413000771 24342856 PMC4080810

[B9] Chatterji P. (2006). Does alcohol use during high school affect educational attainment?: evidence from the national education longitudinal study. Econ. Educ. Rev. 25, 482–497. 10.1016/j.econedurev.2005.05.005

[B10] Chou L.-C. Ho C.-Y. Chen C.-Y. Chen W. J. (2006). Truancy and illicit drug use among adolescents surveyed via street outreach. Addict. Behav. 31, 149–154. 10.1016/j.addbeh.2005.04.011 15905040

[B11] Clark D. B. Pollock N. Bukstein O. G. Mezzich A. C. Bromberger J. T. Donovan J. E. (1997). Gender and comorbid psychopathology in adolescents with alcohol dependence. J. Am. Acad. Child Adolesc. Psychiatry 36, 1195–1203. 10.1097/00004583-199709000-00011 9291720

[B12] Crosnoe R. (2006). The connection between academic failure and adolescent drinking in secondary school. Sociol. Educ. 79, 44–60. 10.1177/003804070607900103 20216913 PMC2834180

[B13] DeSimone J. (2010). Drinking and academic performance in high school. Appl. Econ. 42, 1481–1497. 10.1080/00036840701721554

[B14] Dhalla S. Zumbo B. Poole G. (2011). A review of the psychometric properties of the CRAFFT instrument: 1999–2010. Curr. Drug Abuse Rev. 4, 57–64. 10.2174/1874473711104010057 21466499

[B15] Ellickson P. L. Tucker J. S. Klein D. J. (2003). Ten-year prospective study of public health problems associated with early drinking. Pediatrics 111, 949–955. 10.1542/peds.111.5.949 12728070

[B16] Engberg J. Morral A. R. (2006). Reducing substance use improves adolescents' school attendance. Addiction 101, 1741–1751. 10.1111/j.1360-0443.2006.01544.x 17156173

[B17] Folkehelseinstituttet (2010). The Hordaland Health Study 1997–1999. Bergen. Available online at: http://husk.b.uib.no/

[B18] Hemphill S. A. Heerde J. A. Scholes-Balog K. E. Herrenkohl T. I. Toumbourou J. W. Catalano R. F. (2014). Effects of early adolescent alcohol use on mid-adolescent school performance and connection: a longitudinal study of students in Victoria, Australia and Washington State, United States. J. School Health 84, 706–715. 10.1111/josh.12201 25274170 PMC4196706

[B19] Henry K. L. Thornberry T. P. (2010). Truancy and escalation of substance use during adolescence. J. Stud. Alcohol Drugs 71:115. 10.15288/jsad.2010.71.115 20105421 PMC2815052

[B20] Hishinuma E. S. Chang J. Y. Goebert D. A. Nishimura S. T. Choi-Misailidis S. Andrade N. N. (2006). Substance use as a robust correlate of school outcome measures for ethnically diverse adolescents of Asian/Pacific Islander ancestry. School Psychol. Q. 21:286 10.1521/scpq.2006.21.3.286

[B21] Homel J. Thompson K. Leadbeater B. (2014). Trajectories of marijuana use in youth ages 15–25: implications for postsecondary education experiences. J. Stud. Alcohol Drugs 75, 674–683. 10.15288/jsad.2014.75.674 24988266 PMC4905757

[B22] Horwood L. J. Fergusson D. M. Hayatbakhsh M. R. Najman J. M. Coffey C. Patton G. C.. (2010). Cannabis use and educational achievement: findings from three Australasian cohort studies. Drug Alcohol Depend. 110, 247–253. 10.1016/j.drugalcdep.2010.03.008 20456872

[B23] Janosz M. LeBlanc M. Boulerice B. Tremblay R. E. (1997). Disentangling the weight of school dropout predictors: a test on two longitudinal samples. J. Youth Adolesc. 26, 733–762. 10.1023/A:1022300826371

[B24] Kelly A. B. Evans-Whipp T. J. Smith R. Chan G. C. Toumbourou J. W. Patton G. C.. (2015). A longitudinal study of the association of adolescent polydrug use, alcohol use and high school non-completion. Addiction 110, 627–635. 10.1111/add.12829 25510264 PMC4361375

[B25] Kessler R. C. Adler L. A. Gruber M. J. Sarawate C. A. Spencer T. Van Brunt D. L. (2007). Validity of the world health organization adult ADHD Self-Report Scale (ASRS) screener in a representative sample of health plan members. Int. J. Methods Psychiatr. Res. 16, 52–65. 10.1002/mpr.208 17623385 PMC2044504

[B26] King K. M. Meehan B. T. Trim R. S. Chassin L. (2006). Marker or mediator? the effects of adolescent substance use on young adult educational attainment. Addiction 101, 1730–1740. 10.1111/j.1360-0443.2006.01507.x 17156172 PMC2238681

[B27] Latvala A. Rose R. J. Pulkkinen L. Dick D. M. Korhonen T. Kaprio J. (2014). Drinking, smoking, and educational achievement: cross-lagged associations from adolescence to adulthood. Drug Alcohol Depend. 137, 106–113. 10.1016/j.drugalcdep.2014.01.016 24548801 PMC3964260

[B28] Leach L. S. Butterworth P. (2012). The effect of early onset common mental disorders on educational attainment in Australia. Psychiatry Res. 199, 51–57. 10.1016/j.psychres.2012.03.040 22507527

[B29] Lee S. Tsang A. Breslau J. Aguilar-Gaxiola S. Angermeyer M. Borges G.. (2009). Mental disorders and termination of education in high-income and low-and middle-income countries: epidemiological study. Br. J. Psychiatry 194, 411–417. 10.1192/bjp.bp.108.054841 19407270 PMC2801820

[B30] Lucas C. P. Zhang H. Fisher P. W. Shaffer D. Regier D. A. Narrow W. E.. (2001). The DISC Predictive Scales (DPS): efficiently screening for diagnoses. J. Am. Acad. Child Adolesc. Psychiatry 40, 443–449. 10.1097/00004583-200104000-00013 11314570

[B31] Lundervold A. J. Breivik K. Posserud M.-B. Stormark K. M. Hysing M. (2013). Symptoms of depression as reported by Norwegian adolescents on the short mood and feelings questionnaire. Front. Psychol. 4:613. 10.3389/fpsyg.2013.00613 24062708 PMC3769622

[B32] Oesterle T. S. Hitschfeld M. J. Lineberry T. W. Schneekloth T. D. (2015). CRAFFT as a substance use screening instrument for adolescent psychiatry admissions. J. Psychiatr. Pract. 21, 259–266. 10.1097/PRA.0000000000000083 26164051

[B33] Olsson C. A. Romaniuk H. Salinger J. Staiger P. K. Bonomo Y. Hulbert C.. (2016). Drinking patterns of adolescents who develop alcohol use disorders: results from the victorian adolescent health cohort study. BMJ Open 6:e010455. 10.1136/bmjopen-2015-010455 26868948 PMC4762151

[B34] Perini L. Marti J. (2011). Substance Use And High School Academic Performance. Neuchâtel: Inst. de Recherches Economiques, Univ. de.

[B35] Pilowsky D. J. Wu L.-T. (2013). Screening instruments for substance use and brief interventions targeting adolescents in primary care: a literature review. Addict. Behav. 38, 2146–2153. 10.1016/j.addbeh.2013.01.015 23454877 PMC3623552

[B36] Renna F. (2007). The economic cost of teen drinking: late graduation and lowered earnings. Health Econ. 16, 407–419. 10.1002/hec.1178 17031781

[B37] Roebuck M. C. French M. T. Dennis M. L. (2004). Adolescent marijuana use and school attendance. Econ. Educ. Rev. 23, 133–141. 10.1016/S0272-7757(03)00079-7

[B38] Sabia J. J. (2010). Wastin'away in margaritaville? new evidence on the academic effects of teenage binge drinking. Contemp. Econ. Policy 28, 1–22. 10.1111/j.1465-7287.2008.00120.x

[B39] Skogen J. C. Bøe T. Knudsen A. K. Hysing M. (2013). Psychometric properties and concurrent validity of the CRAFFT among Norwegian adolescents. Ung@ hordaland, a population-based study. Addict. Behav. 38, 2500–2505. 10.1016/j.addbeh.2013.05.002 23770648

[B40] Skogen J. C. Sivertsen B. Lundervold A. J. Stormark K. M. Jakobsen R. Hysing M. (2014). Alcohol and drug use among adolescents: and the co-occurrence of mental health problems. Ung@ hordaland, a population-based study. BMJ Open 4:e005357. 10.1136/bmjopen-2014-005357 25245403 PMC4173106

[B41] StataCorp. (2015). Stata Statistical Software: Release 14. College Station, TX: StataCorp, L.P.

[B42] Staff J. Patrick M. E. Loken E. Maggs J. L. (2008). Teenage alcohol use and educational attainment. J. Stud. Alcohol Drugs 69:848. 10.15288/jsad.2008.69.848 18925343 PMC2583376

[B43] Stiby A. I. Hickman M. Munafò M. R. Heron J. Yip V. L. Macleod J. (2015). Adolescent cannabis and tobacco use and educational outcomes at age 16: birth cohort study. Addiction 110, 658–668. 10.1111/add.12827 25488831 PMC4405050

[B44] Thapar A. McGuffin P. (1998). Validity of the shortened mood and feelings questionnaire in a community sample of children and adolescents: a preliminary research note. Psychiatry Res. 81, 259–268. 10.1016/S0165-1781(98)00073-0 9858042

[B45] Van Ours J. C. Williams J. (2009). Why parents worry: initiation into cannabis use by youth and their educational attainment. J. Health Econ. 28, 132–142. 10.1016/j.jhealeco.2008.09.001 18926578

[B46] Williams J. Powell L. M. Wechsler H. (2003). Does alcohol consumption reduce human capital accumulation? evidence from the college alcohol study. Appl. Econ. 35, 1227–1239. 10.1080/0003684032000090735

[B47] Windle M. (2003). Alcohol use among adolescents and young adults. Population 45, 19–15. 10.1111/j.1532-7795.2006.00131.x

